# Supplementary material for: Novel Driver Strength Index highlights important cancer genes in TCGA PanCanAtlas patients
Source: PeerJ. 2022 Aug 11;10:e13860. doi: 10.7717/peerj.13860 (PMC9375969; doi:10.7717/peerj.13860)
Supplement: Supplemental Information 2 [file peerj-10-13860-s002.zip › COHORTS/genes plots/detailed/2021_8_16_14_9_distribution_events_detailed_14.pdf]

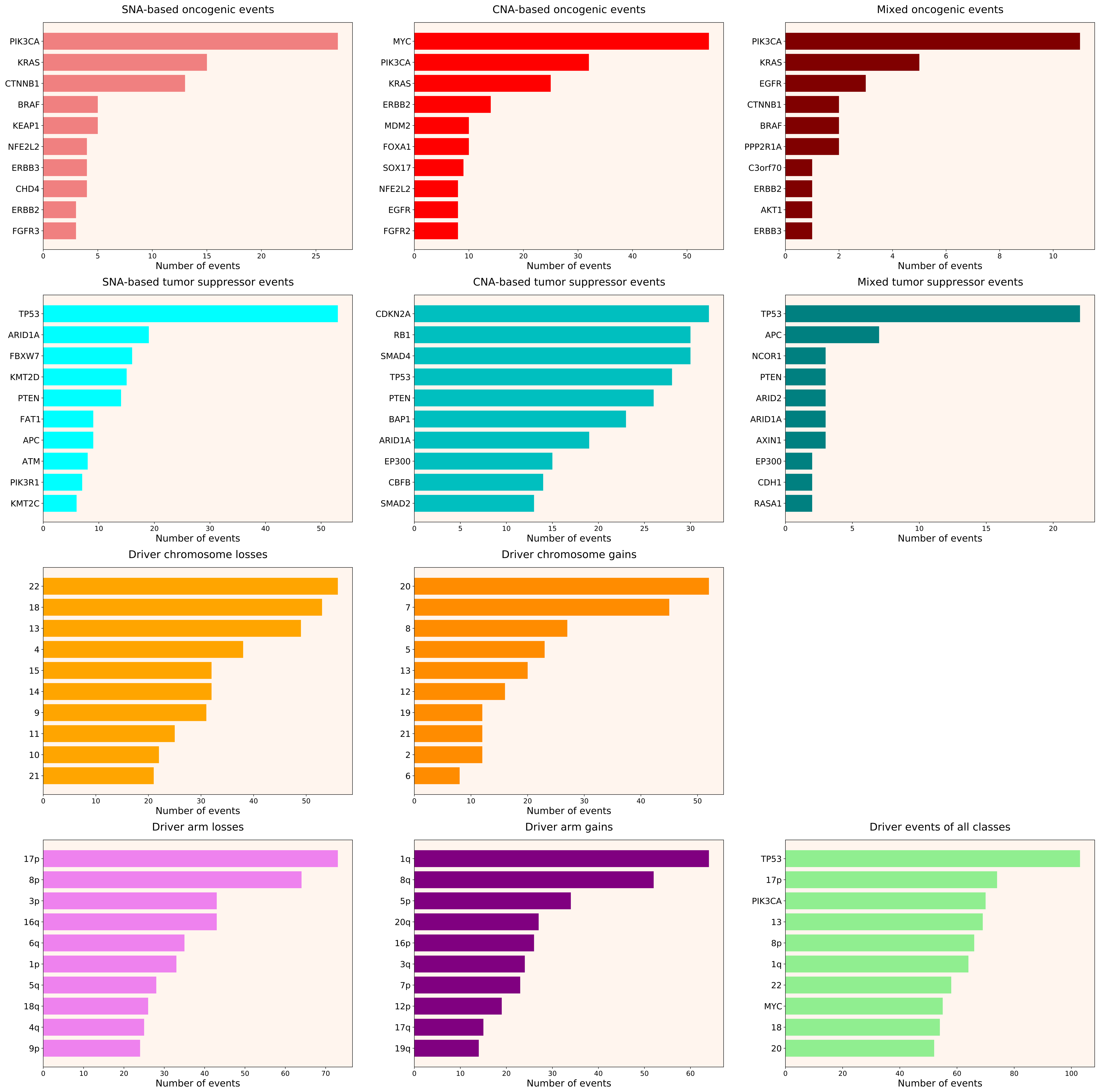
SNA-based oncogenic events
PIK3CA
KRAS
CTNNB1
BRAF
KEAP1
NFE2L2
ERBB3
CHD4
ERBB2
FGFR3
0 5 10 15 20 25
Number of events
CNA-based oncogenic events
MYC
PIK3CA
KRAS
ERBB2
MDM2
FOXA1
SOX17
NFE2L2
EGFR
FGFR2
0 10 20 30 40 50
Number of events
Mixed oncogenic events
PIK3CA
KRAS
EGFR
CTNNB1
BRAF
PPP2R1A
C3orf70
ERBB2
AKT1
ERBB3
0 2 4 6 8 10
Number of events
SNA-based tumor suppressor events
TP53
ARID1A
FBXW7
KMT2D
PTEN
FAT1
APC
ATM
PIK3R1
KMT2C
0 10 20 30 40 50
Number of events
CNA-based tumor suppressor events
CDKN2A
RB1
SMAD4
TP53
PTEN
BAP1
ARID1A
EP300
CBFB
SMAD2
0 5 10 15 20 25 30
Number of events
Mixed tumor suppressor events
TP53
APC
NCOR1
PTEN
ARID2
ARID1A
AXIN1
EP300
CDH1
RASA1
0 5 10 15 20
Number of events
Driver chromosome losses
22
18
13
4
15
14
9
11
10
21
0 10 20 30 40 50
Number of events
Driver chromosome gains
20
7
8
5
13
12
19
21
2
6
0 10 20 30 40 50
Number of events
Driver arm losses
17p
8p
3p
16q
6q
1p
5q
18q
4q
9p
0 10 20 30 40 50 60 70
Number of events
Driver arm gains
1q
8q
5p
20q
16p
3q
7p
12p
17q
19q
0 10 20 30 40 50 60
Number of events
Driver events of all classes
TP53
17p
PIK3CA
13
8p
1q
22
MYC
18
20
0 20 40 60 80 100
Number of events
